# Altered gene expression profiles in the hippocampus and prefrontal cortex of type 2 diabetic rats

**DOI:** 10.1186/1471-2164-13-81

**Published:** 2012-02-27

**Authors:** Omar Abdul-Rahman, Maria Sasvari-Szekely, Agota Ver, Klara Rosta, Bernadett K Szasz, Eva Kereszturi, Gergely Keszler

**Affiliations:** 1 Department of Medical Chemistry, Molecular Biology and Pathobiochemistry, Semmelweis University, Budapest, Hungary; 2 Department of Pharmacology, Institute of Experimental Medicine, Hungarian Academy of Sciences, Budapest, Hungary

## Abstract

**Background:**

There has been an increasing body of epidemiologic and biochemical evidence implying the role of cerebral insulin resistance in Alzheimer-type dementia. For a better understanding of the insulin effect on the central nervous system, we performed microarray-based global gene expression profiling in the hippocampus, striatum and prefrontal cortex of streptozotocin-induced and spontaneously diabetic Goto-Kakizaki rats as model animals for type 1 and type 2 diabetes, respectively.

**Results:**

Following pathway analysis and validation of gene lists by real-time polymerase chain reaction, 30 genes from the hippocampus, such as the inhibitory neuropeptide galanin, synuclein gamma and uncoupling protein 2, and 22 genes from the prefrontal cortex, e.g. galanin receptor 2, protein kinase C gamma and epsilon, *ABCA1 *(ATP-Binding Cassette A1), *CD47 *(Cluster of Differentiation 47) and the *RET *(Rearranged During Transfection) protooncogene, were found to exhibit altered expression levels in type 2 diabetic model animals in comparison to non-diabetic control animals. These gene lists proved to be partly overlapping and encompassed genes related to neurotransmission, lipid metabolism, neuronal development, insulin secretion, oxidative damage and DNA repair. On the other hand, no significant alterations were found in the transcriptomes of the corpus striatum in the same animals. Changes in the cerebral gene expression profiles seemed to be specific for the type 2 diabetic model, as no such alterations were found in streptozotocin-treated animals.

**Conclusions:**

According to our knowledge this is the first characterization of the whole-genome expression changes of specific brain regions in a diabetic model. Our findings shed light on the complex role of insulin signaling in fine-tuning brain functions, and provide further experimental evidence in support of the recently elaborated theory of type 3 diabetes.

## Background

Diabetes mellitus is a chronic and heterogenous metabolic disorder affecting millions of patients worldwide. Type 1 diabetes is characterized by absolute insulin deficiency due to viral or autoimmune destruction of pancreatic beta cells, while the major feature of the more common type 2 variant is obesity-linked impairment of intracellular insulin signaling [1-3]. Apart from its well-known effect on blood sugar levels, insulin is known to regulate the growth, differentiation and metabolism of its target cells at multiple levels [1]. Insulin signaling pathways have been shown to converge on and modulate the transcription of a plethora of genes [2]. In light of this, it is not surprising that gene expression microarrays revealed dramatic alterations in global gene expression profiles of several organs such as skeletal muscles and adipose tissue [3], intestine [4] and the liver [5] both in type 1 and type 2 diabetes.

Although the brain does not count as a classical target organ of insulin, it has recently been shown that this polypeptide hormone plays a crucial role in human neurophysiology, and dysregulation of insulin receptor signaling in various mental illnesses [6].

It has long been known that insulin can pass the blood brain barrier by receptor mediated endocytosis [7]. Moreover, it turned out that several brain regions are capable of producing insulin *in situ *[8]. The insulin receptor and insulin receptor substrate-1 (*IRS1*) are expressed in vegetative nuclei of the hypothalamus, in amygdala, hippocampus and in the neocortex [9]. Based on this expression pattern, cerebral insulin signaling has been implicated in the regulation of neurotransmission, feeding and cognitive functions [10].

Along with leptin, insulin seems to be a negative feedback signal in well-fed state due to its ability to reduce appetite and body weight. It might be assumed that obesity and hyperinsulinism lead to desensitization of insulin receptors situated in the blood brain barrier, giving rise to central insulin resistance [11].

There are several lines of mostly indirect evidence supporting the role of insulin signal transduction in learning and long-term memory. The first observations date back to the famous Rotterdam study, revealing that type 2 diabetes doubled the risk of patients to develop Alzheimer-type dementia, while individuals suffering from type 1 diabetes and receiving insulin therapy had four times the risk [12]. These results were corroborated by more recent studies showing that subjects with elevated body mass index, obesity, insulin resistance and diabetes have an increased risk of dementia and cognitive impairment, suggesting a causal link between decreased insulin secretion and the progression of mental decline [13]. Subsequently, post-mortem brain studies unveiled that cerebral insulin, insulin receptor and *IGF *levels are inversely proportional with the progression of Alzheimer's disease [14]. On the other hand, intranasal and intravenous insulin administration has reportedly improved the cognitive functions of patients suffering from memory disorders, while intracerebroventricular insulin enhanced memory formation in rodents [15,16]. Moreover, intracerebral administration of streptozotocin, a drug known to induce type 1 diabetes by impairing pancreatic β cells when added intravenously, also led to insulin depletion in the brain with subsequent neurodegeneration [17].

The interrelationship between diabetes and Alzheimer's disease seems to be mutual as neurotoxins termed amyloid beta-derived diffusible ligands have been shown to compromise cerebral insulin signaling [18]. On the other hand, oxidative stress elicited by reactive advanced glycation end products (RAGEs) that are characteristic of diabetes might accelerate neuronal damage in memory disorders [19].

Based on these observations, a group of researchers have recently defined Alzheimer's as a neuroendocrine disorder and coined the terms "type 3" or „brain-type" diabetes [20], pointing out that this condition can simultaneously be characterized both by central insulin deficiency and insulin resistance. Their work highlighted the importance of impaired insulin signaling in the dysfunction and apoptotic death of cortical neurons.

Although global transcriptome profiling has already been carried out in Alzheimer's disease [21], according to our best knowledge this is the first study aiming to analyze whole genome gene expression profiles of various cerebral areas in streptozotocin-induced and spontaneously diabetic Goto-Kakizaki rats as model animals for type 1 and type 2 diabetes, respectively. Our results demonstrated an altered expression pattern in the hippocampus and prefrontal cortex of type 2 diabetes model, while no such changes were found in the corresponding brain areas of the type 1 model animals.

## Results

The Agilent rat whole genome custom array encompassed 41,129 different oligonucleotide probes according to the latest annotation of the rat genome. Following normalization and technical screening of raw data, approximately 15-26% of all probes remained. Filtering out genes without significant expression changes resulted in a more drastic reduction of transcript numbers. Statistical analysis and post-screening procedures highlighted spectacular differences in expression profiles of type 2 diabetic brains. Importantly, it turned out that Goto-Kakizaki rats exhibited profound changes in gene expression profiles, while no genes showed significant changes in the transcriptomes of streptozotocin-treated rats versus control animals. Detailed analyses of variations obtained in expression profiles of the studied brain regions of Goto-Kakizaki rats demonstrated large changes in the hippocampus and prefrontal cortex, as 266 versus 147 probes were found to be differentially expressed, respectively, as compared to Wistar controls. Of them, 83 were found in both brain territories. In contrast, only 3 genes with altered expression were identified in the striatum, although they were found in the other two regions as well (Table 1 for detailed gene lists, see Additional File 1). In summary, we obtained a cohort of region-specific or overlapping expression alterations in the Goto-Kakizaki rat model save the striatum that did not show any region-specific patterns at all.

**Table 1:** Number of genes with significant expression changes in specific brain areas of diabetes models vs. control rats.

	Type 2 diabetes model			Type 1 diabetes model		
	Hipp	Pfc	Str	Hipp	Pfc	Str
Statistical analysis	504	232	3	7	0	0
Post-screening	266	147	3	0	0	0
Genes in significant pathways	64	36	0	0	0	0
Genes to be validated*	42	27	0	0	0	0
**Validated genes**	**30**	**22**	0	0	0	0

The table represents significant genes remaining following each stage of the normalization-evaluation procedure. For details, see Methods and Results sections. Abbreviations used: Hipp: hippocampus; Pfc: prefrontal cortex; Str: striatum.

* Reduction was due to technical criteria of the TaqMan RT-PCR system (only genes with commercially available TaqMan probes could be validated).

Next, we wished to assign biological relevance to our gene lists by ordering them in biochemical pathways. The Biological Process domain of the Gene Ontology database provided the most extensive pathway assignment. 64 genes from the hippocampus and 36 from the prefrontal cortex were found to be members of certain pathways (Table 1).

**Figure 1:**
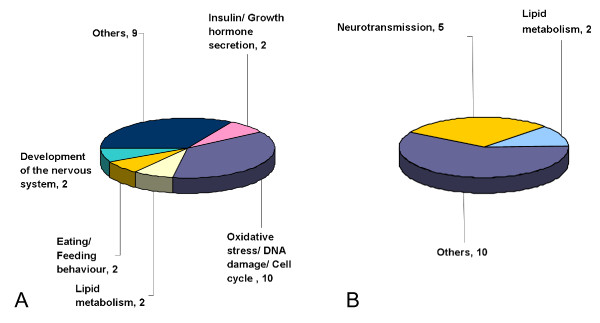
**Distribution of significant genes by functional categories in the hippocampus (A) and in the prefrontal cortex (B) of Goto-Kakizaki rats**. The number of significantly altered pathways is also indicated in each category.

Finally, gene expression changes fulfilling the criteria of mathematical-statistical selection and pathway analysis were validated by real time PCR using TaqMan Low Density Arrays. It should be noted that only genes with commercially available TaqMan probes could be analyzed. Therefore, 42 out of the 64 hippocampal and 27 out of the 36 prefrontal genes were subject to validation. Finally, 30 genes from the hippocampus (71%) and 22 genes from the prefrontal cortex (82%) were validated (Table 2; for detailed gene lists, see Additional File 2). According to our results, 9 genes showed changes both in the hippocampus and in prefrontal cortex in the type 2 diabetes model (for a detailed list, see Additional File 2). Finally, pathway analysis revealed that most genes with altered expression patterns in the hippocampus are involved in oxidative stress and DNA damage signaling, cell cycle regulation, development and lipid metabolism of the central nervous system as well as in the regulation of feeding behavior (Table 2 and Figure 1).

**Table 2:** List of significant pathways in the hippocampus of type 2 diabetic rats.

GO Biological processes	HIPPOCAMPUS	Validated	Not validated
Insulin/GH secretion	GO:30073: insulin secretion	**Gal**	
	GO:30252: growth hormone secretion	**Gal**	

Oxidative stress DNA damage cell cycle	GO:6950: response to stress	**Gal**	
	GO:305: response to oxygen radical	Cxcl4(Pf4)	
	GO:303: response to superoxide	Akap3	
	GO:302: response to reactive oxygen species	**Gal**	Nudt15_predicted
	GO:15992: proton transport	Ucp2	
	GO:6977: DNA damage response, signal transduction by p53 class mediator resulting in cell cycle arrest	Ptprv	
	GO:42770: DNA damage response, signal transduction	Ftcd	
	GO:7346: regulation of progression through mitotic cell cycle	Snf1lk	
	GO:6269: DNA replication, synthesis of RNA primer	NM_001008768 (Prim1)	
	GO:7089: traversing start control point of mitotic cell cycle	Cdk10	

Lipid metabolism	GO:1573: ganglioside metabolism	Gm2a	
	GO:6695: cholesterol biosynthesis	Acaa2	Acaa2

Eating/feeding behavior	GO:7631: feeding behavior	**Gal**, Agrp	
	GO:42755: eating behavior	Agrp	Stat3

Development of the nervous system	GO:7399: nervous system development	**Gal, Mobp, Mobp, Cntn3**	Ednrb, RGD1311340_predicted, Stat3, XM_242005
	GO:7422: peripheral nervous system development	Sncg	Ednrb

Others	GO:50776: regulation of immune response	**Gal**, Il22ra2	
	GO:6952: defense response	Mx2	
	GO:7194: negative regulation of adenylate cyclase activity	**Grm2**	
	GO:6032: chitin catabolism	Chi3l1	
	GO:42572: retinol metabolism	Retsat	
	GO:45123: cellular extravasation	Itgam	
	GO:19637: organophosphate metabolism	Pter	
	GO:6928: cell motility	Akap3, **Grm2**	Stat3
	GO:9615: response to virus	Mx2, Oas1	XM_215121

Data were obtained using the GO pathway analysis software; specific GO pathway identification numbers are provided in the second column. "Validated genes" were confirmed by quantitative PCR analysis. Validated genes found in more than 2 significant pathways are shown in bold.

Regarding the prefrontal cortex, perturbed expression of a set of neurotransmission and lipid metabolism related genes has been unveiled with significant overlap with the hippocampal alterations (Table 3 Additional File 2 and Figure 1). These findings seem to be consistent with functional cerebral impairments described in diabetic individuals such as cognitive deficit, increased appetite and food ingestion, and development of depression [22]. It would be of importance to clarify whether genes with altered expression patterns are controlled by insulin-dependent transcription factors such as members of the forkhead (*FOXO*) family [23].

**Table 3:** List of significant pathways in the prefrontal cortex of type 2 diabetic rats.

GO Biological processes	PREFRONTAL CORTEX	Validated	Not validated
neurotransmission	GO:7611: learning and/or memory	**Galr2, Prkcc, Gm2a**	
	GO:7268: synaptic transmission	**Galr2, Prkcc, Grm2**	
	GO:1507: acetylcholine catabolism in synaptic cleft	Colq	
	GO:1504: neurotransmitter uptake	Slc17a6	
	GO:17158: regulation of calcium ion-dependent exocytosis	Trpv6	

lipid metabolism	GO:1573: ganglioside metabolism	Gm2a	
	GO:45332: phospholipid translocation	Abca1	

others	GO:9649: entrainment of circadian clock	Bhlhb2	
	GO:8228: opsonization	Cd47	
	GO:6032: chitin catabolism	Chi3l1	
	GO:6547: histidine metabolism	Ftcd	
	GO:7497: posterior midgut development	Ret	
	GO:30277: maintenance of gastrointestinal epithelium	Tff1	
	GO:6936: muscle contraction	**Galr2**, Lsp1	Sgca_predicted
	GO:19882: antigen presentation	NM_001008842, RT1-Aw2 (Y13890)	
	GO:9615: response to virus	Oas1	XM_215121
	GO:7635: chemosensory behavior	Prkcc, Prkce	Prkce

Data were obtained using the GO pathway analysis software; specific GO pathway identification numbers are provided in the second column. "Validated genes" were confirmed by quantitative PCR analysis. Validated genes found in more than 2 significant pathways are shown in bold.

## Discussion

Insulin regulates gene expression via a set of transcription factors including the *FOXO *family [24]. As insulin and its receptors are both known to be expressed and to govern important functions in the brain, it seemed reasonable to search for altered gene expression patterns in animal models of type 1 and type 2 diabetes characterized by absolute or relative insulin deficiency. Here we demonstrated a substantial difference in the gene expression pattern of type 2 diabetic rats vs. control animals. The genetically determined, spontaneously diabetic Goto-Kakizaki rats exhibited profound gene expression alterations suggesting that long-standing impairment of insulin signaling has a well detectable effect on the central nervous system. On the other hand, we could hardly detect any alterations in the streptozotocin-induced diabetic animal model (Table 1), suggesting that acute insulin deficiency and/or elevated blood sugar levels do not influence significantly the cerebral gene expression pattern, or at least it is undetectable four weeks after the streptozotocin treatment in a microarray based experiment. It is tempting to speculate that streptozotocin-induced diabetic rats might successfully compensate peripheral insulin deficiency by increased cerebral insulin production. However, this presumption seems to contradict the fact that activation of the *ins2 *gene was not detected - maybe due to low sensitivity of the whole genome custom array.

Three main brain regions have been studied here: the prefrontal cortex and hippocampus were analyzed due to their well-known roles in learning and memory formation, while the striatum seemed to be an easily dissectable control region where no insulin action had been presumed. It is also interesting to note that streptozotocin-treted rats exhibited some gene expression alterations in the hippocampus only. These observations are in a good agreement with the findings of Agrawal et al., showing that insulin and its receptor are mostly expressed in this brain region, and intracerebroventricular administration of streptozotocin induced memory deficit in rats [25].

Streptozotocin has been proven to induce insulin deficiency and hyperglycemia (≥ 15 mM) within 72 hours in treated animals, and they were alive for 4 weeks following beta-cell destruction. In our opinion, this time window should have been enough to alter gene expression profiles in the brain as there are several reports highlighting the early effects of streptozotocin on gene expression in various organs [26]. The major drawback of the global microarray method is its minor sensitivity compared to that of TaqMan-based quantitative reverse transcription PCR assays. However, the high RT-PCR validation rate of microarray data in Goto-Kakizaki rats (71% in the hippocampus and 82% in the prefrontal cortex, respectively) convinced us of the reasonably good reliability of the chip hybridization technique. Theoretically, some minor gene expression alterations in the brains of type 1 diabetic model animals might have been left undetected by the chip hybridization technique, therefore, we are committed to validate the "non-changed" status of a set of genes which were significantly altered in type 2 diabetic animals using open-array real-time PCR assays.

Analyzing the specific genes, the mRNA levels of galanin, an inhibitory neuropeptide with pleiotropic roles were substantially upregulated in the hippocampus. Notably, galanin were identified in almost all perturbed pathways of the hippocampus (Table 2). Our results corroborated the findings of Mei et al. who detected elevated galanin expression in the celiac ganglion in diabetic rats [27]. Intracerebroventricular administration of galanin or its overexpression in transgenic mice was shown to compromise hippocampus-dependent learning processes [28,29]. Galanin has been proposed to play a role in depression-like behavior [30]. On the other hand, improvement of cognitive functions has been reported in animals treated with galanin receptor antagonists [28]. As cerebral insulin deficiency presents with similar symptoms, it is tempting to speculate that impairment of cerebral functions in diabetes might be mediated at least in part by elevated galanin levels. This assumption is supported by the fact that plasma galanin levels have been found to be significantly elevated in patients with type 2 diabetes [31], and increased plasma galanin levels were measured following oral glucose load in a healthy population [32]. If we managed to find a causal relationship between cerebral insulin deficiency and galanin overexpression, we might be able to ameliorate cerebral symptoms of diabetes via pharmacological modulation of galanin receptors and to slow down the progression of type 3 diabetes [20].

The role of galanin receptors is also highlighted by our results which demonstrated altered galanin receptor 2 expression levels in the prefrontal cortex (Table 3). Type 2 galanin receptors are mostly expressed in the perikaryon of neurons, mediating calcium signals and promoting the survival of neurons [33], and their stimulation reportedly elicited antidepressive effects [34].

Apart from galanin and its receptor, there are several other validated genes as well, which have already been implicated in the pathogenesis of both diabetes and psychiatric disorders in some respect. For instance, *Chi3l1 *(*YKL-40*, chitinase 3-like 1) has recently been shown to represent an obesity-independent novel marker of type 2 diabetes [35]. On the other hand, *Chi3l1 *has been regarded as a schizophrenia susceptibility gene, a mediator of stress-induced cellular responses [36]. *SNCG *(synuclein gamma) has recently been termed an adipocyte-neuron gene that is coordinately expressed with leptin in human obesity and might promote adipocyte differentiation [37]. Apart from its well-known role in the development of neurodegenerative diseases [38], *SNCG *has also been implicated in depression [39], dopamine release [40] and as an interacting partner of the dopamine transporter in rats [41].

Perturbation of brain signaling pathways could also be a very important hallmark of type 2 diabetes. Here we identified three genes of cerebral signaling (protein kinase C gamma and epsilon, and the *RET *tyrosine kinase) with altered cerebral expression profiles in Goto-Kakizaki rats. They have been shown to play a pathophysiological role in brain dysfunction previously. For instance, expression of the neuron-specific gamma isoform of protein kinase C (*Prkcc*) that has been implied in the regulation of learning and memory formation (Additional File 2) was more than twofold upregulated in the prefrontal cortex of Goto-Kakizaki rats (Additional File 2). Schlaepfer et al. demonstrated that certain polymorphisms of the *Prkcc *gene are associated with behavioral disinhibition and attention deficit hyperactivity disorder (ADHD) in humans, while *PKC-gamma *deficient mice exhibited impulsivity, anxiety and increased ethanol consumption [42]. Importantly, the epsilon isoform of *PKC *(*Prkce*) is also overexpressed in the type 2 diabetic model (Additional File 2). This kinase is reportedly involved in neuronal ion channel activation, apoptosis and insulin exocytosis. Recently, *Prkce *has been implicated in the loss of insulin secretory responsiveness during the development of type 2 diabetes [43], while others highlighted its role in the pathomechanism of drug dependence and addiction [44]. Shelton et al. revealed decreased *Prkce *protein levels in post mortem brain specimens of patients with major depression [45]. Finally, we demonstrated changes in the expression level of the *RET *protooncogene, a receptor tyrosine kinase containing cadherin-like repeats in its extracellular domain, that plays a pivotal role in neural crest development. Mutations in this gene might elicit multiple endocrine neoplasia type 2B with diabetes [46]. Interestingly, *RET *activity has been shown to modulate and shape the brain dopaminergic systems which are known mediators of several personality traits [47].

As far as the theory of type 3 diabetes is concerned, our microarray data revealed a couple of genes which might provide a link between diabetes and neurodegeneration. Apart from the already mentioned synuclein gamma, uncupling protein 2 (*UCP2*), the ABC-transporter *ABCA1 *and the cell surface antigen *CD47 *should also be mentioned in this context. *UCP2*, a well-known inner mitochondrial membrane protein, responsible for energy dissipation and heat production, has been found to associate with obesity, diabetes and regulation of insulin secretion [48]. On the other hand, the *UCP2 *gene is induced in a ghrelin-dependent fashion and protects from neurodegeneration [49]. *UCP2 *expression was significantly downregulated in the hippocampus of our type 2 diabetic rat model (Additional File 1), implying that its neuroprotective effect might be absent from the diabetic brain.

Mutations in the cholesterol efflux pump *ABCA1 *have been associated with Tangier's disease. Beyond that, *ABCA1 *has been implicated in insulin secretion from pancreatic beta cells [50], and some single nucleoide polymorphisms (SNPs) of this gene have been demonstrated to associate with dementia (rs2230805) [51] and Alzheimer's disease (rs1800977 and rs2422493) [52]. We found significant downregulation of *ABCA1 *levels in the prefrontal cortex of Goto-Kakizaki rats (Additional File 2); hence it seems logical to assume that elevated cytosolic cholesterol levels might impair the viability of neurons via affecting membrane fluidity.

The gene for *CD47 *encodes a membrane protein which is involved in the increase in intracellular calcium concentration that occurs upon cell adhesion to the extracellular matrix. There is ample evidence supporting the role of *CD47 *in pancreatic insulin secretion [53]. Moreover, *CD47 *has been shown to interact with amyloid beta peptide in Alzheimer's disease [54]. We measured elevated *CD47 *mRNA levels both in the hippocampus and in the prefrontal cortex of type 2 diabetes model animals, providing a plausible link between central insulin resistance and Alzheimer-type neurodegeneration.

## Conclusion

In conclusion, our study shed light on the seminal role of insulin in maintaining the functions of the central nervous system by unveiling characteristic perturbations in cerebral gene expression profiles in type 2 diabetic rats. We identified several cerebral expression changes in genes which were previously assumed to play a role in pancreatic insulin secretion, implying that these genes might mediate insulin production and exocytosis in the brain as well. Our results should prompt further investigations to decipher insulin signaling pathways in the brain and a detailed analysis of the transcriptional regulation of diabetes-associated genes having been identified in this study.

## Methods

### Animals

Experiments were performed on ten-week old male rats (weighing 286 ± 60 g). Streptozotocin-treated inbred white Wistar rats were used as model animals for type 1 diabetes, and Goto-Kakizaki rats were the polygenic non-obese models of type 2 diabetes [55]. Wistar rats at 6 weeks of age, weighing approximately 170 g, were injected with 65 mg/body mass kg streptozotocin intravenously. The development of diabetes was confirmed by elevated fasting blood sugar levels (≥ 15 mM measured 72 hrs following the injection), and the streptozotocin-treated rats were sacrificed by cervical dislocation 4 weeks after the injection. Diabetic animals as well as their age- and body mass matched Wistar controls and age-matched Goto-Kakizaki rats were kept on normal chow. All experimental protocols were in accordance with the guidelines of the Committee on the Care and Use of Laboratory Animals of the Council on Animal Care at the Semmelweis University, Budapest, Hungary (ethical permission No.: TUKEB 99/94).

### Tissue harvesting

9 animals from each group at 10 weeks of age were anaesthetized with phenobarbital and killed by decapitation. The brain was removed and the striatum, hippocampus and prefrontal cortex were dissected. Samples from 3-3 identically treated animals were pooled. That means, 3 biological parallels were prepared from each brain region of type 1 or type 2 diabetic and control animals, amounting to a total of 27 different pooled samples. Excised tissue samples were immediately fixed in RNAlater RNA stabilization reagent (Qiagen).

### Sample preparation and oligonucleotide microarray hybridization

Total RNA was extracted from samples by homogenization using the RNeasy Kit (Qiagen), according to the manufacturer's instructions. RNA integrity and purity were checked both by agarose gel electrophoresis and with an Agilent 2100 Bioanalyzer. Samples of acceptable quality fulfilled the following criteria: OD_260/280 _> 1.8, OD_260/230 _> 1.8 and RIN > 7. Reverse transcription was performed using 1000 ng of total RNA from each sample. Labeling of single-stranded cRNA, hybridization and scanning were carried out at the Microarray Core Facility of the Department of Genetics, Cell- and Immunobiology of Semmelweis University, using Agilent's One-Color Microarray-Based Gene Expression Analysis Protocol, Version 5.5 (G4140-90040). Labeling of samples was performed with Agilent's Low RNA Input Linear Amplification Kit PLUS assay using the Cy3 dye. Dye incorporation was controlled by a Nanodrop spectrophotometer; all samples were labeled with an efficiency of 10.2 - 17.5 pmol Cy3/μg cRNA. 1650 ng of cRNA were hybridized to Agilent's Rat Whole Genome Custom Arrays. Arrays were run on all 27 biological samples. Hybridized arrays were imaged with Agilent's Microarray Scanner, Agilent Feature Extraction Software version 9.1 in the extended dynamic range at 100% and 10% laser beam intensities at a resolution of 5 μm.

### Data analysis

Data analysis was performed using the GeneSpring GX software (Agilent Technologies, version 7.3). For normalization, the samples were grouped according to brain areas. In this way, gene expression data from treated samples in groups were normalized to the median of control samples of each group. As quality control, genes with poor hybridization signals (flag screening) and those with unaltered expression (not showing a minimum of 2-fold difference between their maximal and minimal expression levels under any conditions) were excluded from subsequent analysis. Statistical analysis of data obtained from the normalization and screening procedures was performed to select probes with at least a twofold, statistically significant expression alteration in type 1 or type 2 diabetic animals compared to Wistar controls using Welch's *t*-test supplemented with the Benjamini-Hochberg multiple correction test with a p = 0.05 cutoff. Finally, a post-screening procedure was implemented to exclude false positive probes, i.e. signals with "absent" flag in at least 2 out of 3 biological replicates, and those with raw intensity signals less than 100 arbitrary units.

The Gene Ontology database (URL: http://www.geneontology.org)was used to assign biological relevance to our data and to identify genes by ordering them in relevant biochemical pathways. Biochemical pathways were regarded significantly altered if they comprised a significant number of genes from our lists (p < 0.05).

### Validation by real-time PCR

Genes that fulfilled the criteria of technical, statistical and pathway analyses were validated by the quantitative reverse transcription PCR-based TaqMan Low Density Array (Applied Biosystems) system, according to the manufacturer's protocol. cDNA samples for this test were synthesized from the same RNA samples that had been prepared for microarray hybridization. Relative gene expression data were obtained using the 2(-Delta Delta C_T_) method described by Livak and Schmittgen in detail [56].

Briefly, six genes were selected as potential housekeeping (internal control) genes for normalization of RT-PCR data [histone deacetylase 3 (*Hdac3*), ATP-citrate lyase (*Acly*), beta-actin (*Actb*), beta-2 microglobulin (*B2m*), TATA box binding protein (*Tbp*), 18S ribosomal RNA (*18S*)]. By cross-checking their relative expression levels and scattering scores, we chose the following 3 genes with most stable and constant expression: *Hdac3*, *Tbp *and *B2m*. The expression of all target genes was normalized to the mean of the expression of the housekeeping genes (relative quantification). Cycle threshold (C_T_) values were set in the exponential range of the amplification plots using the 7300 System Sequence Detection Software 1.3. ΔΔC_T_-values corresponded to the difference between the C_T_-values of the genes examined and those of the arithmetical mean of the expression of the 3 housekeeping calibrator (internal control) genes. Relative expression levels of genes were calculated and expressed as 2^-ΔΔCT^. Finally, the Mann-Whitney test (p < 0.01) was used for statistical analysis of qRT-PCR data.

### Data deposition

The data discussed in this publication have been deposited in NCBI's Gene Expression Omnibus (Edgar et al., 2002) and are accessible through GEO Series accession number GSE34451 (http://www.ncbi.nlm.nih.gov/geo/query/acc.cgi?acc=GSE34451).

## Authors' contributions

OA performed the data and statistical analysis; MS conceived of the study, participated in its design and coordinated the study; AV and KR participated in the design of the study and selection of animals; BKS performed brain dissections and tissue harvesting, EK participated in mRNA preparation; GK participated in study design and drafted the manuscript. All authors read and approved the final manuscript.

## Supplementary Material

Additional file 1 **List of differentially expressed genes in the brain areas of Goto-Kakizaki rats**. Genes are ordered according to their fold expression changes. Genes are identified both by gene name, Genbank accession number and gene symbol. The file contains four table sheets displaying genes with significantly altered expression levels (more than twofold or less than 0.5 fold) in the hippocampus only ("Hippocampus", 180 genes), in the prefrontal cortex only ("Prefrontal cortex", 61 genes), both in hippocampus and prefrontal cortex ("Hipp&Pfc", 83 genes) and both in hippocampus, prefrontal cortex and striatum ("Hipp&Pfc&Str", 3 genes), respectively. In the corporate lists genes are ordered according to their fold expression changes observed in the hippocampus. Click here for file

Additional file 2 **List of validated genes in the brain areas of Goto-Kakizaki rats**. Genes are shown in alphabetical order of gene symbols. Genes are identified both by gene name, Genbank accession number and gene symbol. The file contains three table sheets displaying genes with RT-PCR validated, significantly altered expression levels (more than twofold or less than 0.5 fold) in the hippocampus only ("Hippocampus", 30 genes), in the prefrontal cortex only ("Prefrontal cortex", 22 genes), both in hippocampus and prefrontal cortex ("Hipp&Pfc", 9 genes), respectively. In the corporate lists genes are ordered according to their fold expression changes observed in the hippocampus. Click here for file
